# Prediction of early recovery in patients with acute peripheral facial paralysis using serial electroneuronography

**DOI:** 10.1371/journal.pone.0337613

**Published:** 2025-12-02

**Authors:** Yong Seok Jo, Seung Jae Lee, Hyun Jin Lee, Jeon Mi Lee

**Affiliations:** 1 Department of Otorhinolaryngology-Head and Neck Surgery, Dongtan Sacred Heart Hospital, Hallym University College of Medicine, Hwaseong, Korea; 2 Department of Otorhinolaryngology, Ilsan Paik Hospital, Inje University College of Medicine, Goyang, Korea; 3 Department of Otorhinolaryngology-Head and Neck Surgery, Incheon St. Mary’s Hospital, College of Medicine, The Catholic University of Korea, Seoul, Korea; Ohio State University, UNITED STATES OF AMERICA

## Abstract

**Objectives:**

This study aimed to determine the preferred timing and measurement sites for electroneuronography (ENoG) to predict early recovery from acute peripheral facial paralysis.

**Methods:**

We retrospectively evaluated 42 patients with acute peripheral facial paralysis who received standard treatment with oral corticosteroids. The severity of facial paralysis was assessed at the initial visit and after 1 month using the House–Brackmann grading system. Patients were classified into recovery and non-recovery groups according to changes in the grade. ENoG was performed at the initial visit and after 2 weeks. ENoG amplitudes of four facial muscles (frontalis, nasalis, orbicularis oculi, and orbicularis oris) at the initial visit and after 2 weeks, as well as age, sex, affected side, and diagnosis, were compared between the two groups.

**Results:**

No differences were observed in degeneration ratios across all subsites in the initial ENoG, which can be explained by the fact that Wallerian degeneration is not yet complete at this early stage. However, the second ENoG, performed after degeneration had progressed, showed significant differences across all subsites. Binary logistic regression analysis revealed that the degeneration ratio of the orbicularis oris muscle was the best predictor of early recovery (odds ratio, 0.961; p = 0.014). Receiver operating characteristic curve analysis also revealed that the degeneration ratios of all subsites measured in the second ENoG were useful in predicting early recovery, with the highest possibility at the orbicularis oris muscle (area under the curve = 0.789). When the degeneration ratio exceeded 60% in all subsites in the second ENoG, a favorable prognosis was not expected.

**Conclusion:**

This study provides the preferred testing time and measurement sites for ENoG to predict early recovery from facial paralysis. Given the personal and social impact of facial paralysis, predicting early recovery is crucial for reassuring patients, providing better treatment, and encouraging early reintegration into society.

## Introduction

Acute peripheral facial paralysis is the most common cranial nerve disorder, with an incidence of 20–30 cases per 100,000 people annually [1–4]. Facial paralysis is closely related to extroversion, conscientiousness, emotional stability, and depression [5], and its impact is greater in modern societies where appearance is valued. Therefore, facial paralysis can have a significant impact not only on individuals but also on socioeconomic aspects such as employment and medical costs [6,7].

Initial diagnosis and evaluation of facial paralysis are performed through a physical examination. Several standardized scales, the most common being the House–Brackmann and Sunnybrook grading systems, have been developed to assess the degree of facial paralysis. However, these are subjective tests in which inter- and intra-test variabilities exist [8]. Electrophysiological approaches have been introduced to quantify the degree of facial paralysis objectively. These include electroneuronography (ENoG), electromyography, blink reflex, nerve excitability tests, and maximal stimulation tests. Among these, ENoG is currently the most widely used tool as it is noninvasive, can quantify the degree of damage without the intervention of an examiner, and provides reliable results within 3–21 days of onset, making it useful in the early stages of the condition [9,10].

The standard treatment for peripheral facial paralysis is systemic steroids alone or in combination with antiviral therapy [11–14]. However, severe cases may require surgical intervention, which is reportedly more effective than systemic steroid therapy [15,16], and it should be performed within 14 days of onset. The middle cranial fossa approach, which better exposes the labyrinthine segment, has been reported to be more effective than the transmastoid approach [17]. Nonetheless, surgical interventions carry a risk of serious complications such as cerebral hemorrhage, infections, seizures, and cerebrospinal fluid leakage, and even the intervention itself can cause additional nerve damage [4,17]. This could be a huge burden for both clinicians and patients when deciding the treatment plan in the early stages. This emphasizes the importance of an accurate assessment method capable of precisely predicting the extent of damage and the potential for recovery.

ENoG, a noninvasive test that can be easily performed in outpatient settings for patients in the early stages of paralysis, plays an important role in predicting outcomes and guiding treatment decisions. However, its results are largely influenced by the timing of the test and the subsite examined. Although previous studies have evaluated the timing of ENoG, to our knowledge none have systematically compared degeneration patterns across multiple subsites as in the present study.

In this study, we aimed to analyze the results of two ENoG tests conducted between 3 days and 3 weeks (once during the first week and later during the second to third weeks) after the onset of acute peripheral facial paralysis to evaluate the impact of degeneration ratios on the prediction of early recovery. This study aims to add clarity to the usefulness and timing of ENoG in the ability to provide patients with an accurate prognosis to help the clinician and the patient guide treatment decisions including surgery.

## Materials and methods

### Study participants

This was a single-center retrospective analysis of adult patients who visited the hospital between 01/01/2021 and 31/12/2023. Patients with tumors or trauma and those who underwent vaccination were excluded. Patients who underwent procedures such as botulinum toxin injection or surgical treatment, which made it difficult to assess spontaneous recovery, were excluded. Patients with bilateral or recurrent facial paralysis were also excluded. Consequently, the study population was limited to patients with Bell’s palsy (BP) or Ramsay–Hunt Syndrome (RHS), the most common causes of peripheral facial paralysis.

All patients received standard treatment for acute peripheral facial paralysis, consisting of high-dose oral corticosteroids (1 mg/kg prednisolone, up to a maximum of 60 mg, once daily) for 10–14 days. Antiviral agents were administered to patients with RHS. ENoGs were performed at the initial visit and repeated at the end of the treatment period, that is, 10–14 days after the first visit. Patients who underwent ENoG twice within the effective time window (3–21 days from onset) were included.

**Fig 1:**
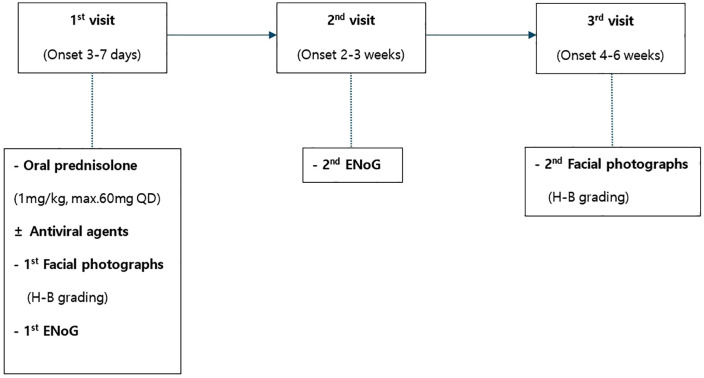
Flow diagram of the study.

A schematic flow diagram of the study design is shown in **Fig 1**. This study was approved by the Institutional Review Board of Inje University Ilsan Paik Hospital (IRB File No. 2024-03-020). The institution waived the requirement for written consent owing to the retrospective design. The data were accessed for research purposes from 12/04/2024–02/01/2025. Authors had no access to information that could identify individual participants during or after data collection.

### Assessment of the severity of facial paralysis and patient classification

Facial photographs of seven expressions were taken at the initial visit and 4–6 weeks after onset. The seven expressions were the resting state, saying “e,” saying “o,” looking upward to create wrinkles on the forehead, closing the eyelid gently, closing the eyelid tightly, and blowing up a balloon. Two board-certified otolaryngologists with subspecialty training in neurotology independently evaluated the severity of facial paralysis using the House–Brackmann grading system, and interobserver agreement was ensured by using the average of their scores.

Patients were classified into two groups based on the changes in their grades. The recovery group included patients whose grades improved by 2 or more or returned to grade 1 (normalization). Patients who did not meet these criteria were classified into the nonrecovery group.

### ENoG measurement

**Fig 2:**
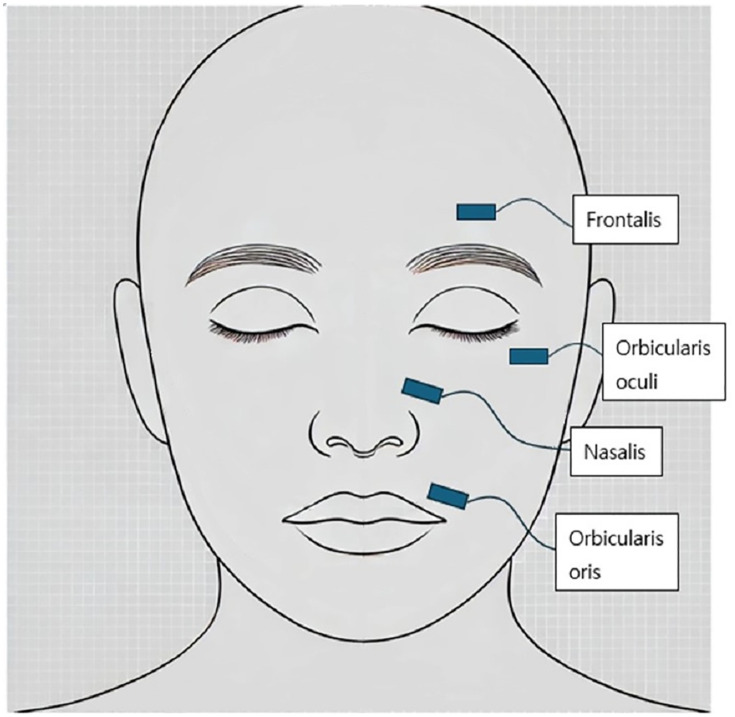
Location of placing the recording electrodes for electroneuronography.

ENoG (Nicolet VikingQuest, Natus, USA) was performed twice within 3–21 days of onset. Recording electrodes were placed on the frontalis, orbicularis oculi, nasalis, and orbicularis oris muscles **(Fig 2)**. Electrical stimulation was applied to the main trunk, and the amplitudes of compound muscle action potentials (CMAPs) were measured at each subsite.

The ratio of CMAPs on the paralyzed side to those on the healthy side reflected the degree of facial nerve amplitude ratio on the paralyzed side (amplitude ratio = CMAPs on the paralyzed side/CMAPs on the healthy side × 100%). The value obtained by subtracting the amplitude ratio from 100% is defined as the degeneration ratio (degeneration ratio = 100% – amplitude ratio).

### Statistical analysis

Statistical analyses were performed using SPSS, version 24 (IBM Corp., Armonk, New York, USA). Continuous variables between the two groups were compared using the independent t-test and three or more groups were compared using the one-way analysis of variance. The chi-square and Fisher’s exact tests were used to assess differences in proportions between the groups. Binary logistic regression analysis was performed to identify predictors of early recovery, adjusting for potential confounders such as age, sex, and diagnosis type (Bell’s palsy or Ramsay Hunt syndrome). Receiver operating characteristic (ROC) curve analysis was performed to assess the degree to which ENoG measurements could predict early recovery. Statistical significance was set at p < 0.05.

## Results

### Study population

A total of 112 patients presented with facial paralysis during the study period. Patients with recurrent episodes, post-vaccination onset, central causes, chronic paralysis, trauma, middle ear cholesteatoma, post-surgical complications, or missed follow-ups were excluded. Following these exclusions, 93 patients remained, and 42 of them who underwent two consecutive ENoGs within 3–21 days after onset were included in the final analysis.

The mean age of the included patients was 48.8 ± 18.0 years (13–92 years). The study population comprised 23 males (54.8%), and the right side was the most affected (n = 28, 66.7%). Overall, 35 patients were diagnosed with BP (83.3%) and 7 were diagnosed with RHS. The first and second ENoGs were conducted, on average, 4.8 ± 2.5 and 14.8 ± 3.0 days after onset, respectively.

Notably, 30 patients (71.4%) were classified into the recovery group and 12 (28.6%) were classified into the non-recovery group. The mean ages of the two groups were 47.1 ± 18.0 and 53.2 ± 20.2 years, respectively, with no statistical difference (p = 0.331). The male-to-female ratio was comparable between the groups (p = 0.769). The recovery group included 26 patients with BP and 4 with RHS, whereas the non-recovery group included 9 patients with BP and 3 with RHS. The recovery group had a relatively higher proportion of patients with BP, whereas the non-recovery group had a higher proportion of those with RHS; however, the difference was not statistically significant (p = 0.387). The demographic data of patients are presented in **Table 1**.

**Table 1:** Demographics of the patients.

	Total	Recovery group	Nonrecovery group	P-value
Number (%)	42	30 (71.4%)	12 (28.6%)	
Age (mean±SD), years	48.8 ± 18.0	47.1 ± 18.0	53.2 ± 20.2	0.331^a)^
Sex (M:F), n	23:19	16:14	7:5	0.769^b)^
Site (R:L), n	28:14	20:8	10:4	1.0^c)^
Diagnosis (BP:RHL), n	35:7	26:4	9:3	0.387^c)^
First ENOG (mean±SD), days	4.8 ± 2.5	4.8 ± 2.4	4.8 ± 2.8	0.924^a)^
Second ENOG (mean±SD), days	14.8 ± 3.0	15.2 ± 2.6	13.8 ± 3.9	0.155^a)^

BP, Bell’s palsy; RHS, Ramsay-Hunt syndrome; ENoG, Electroneuronography; SD, standard deviation. ^a)^by Independent t-test, ^b)^by chi-square test, ^c)^by Fisher exact test.

### Comparison of facial nerve degeneration ratios

Both the recovery and non-recovery groups showed increased degeneration ratios from the initial test to the second test. However, there were no differences between the groups in the initial ENoG, whereas significant differences were observed in the second test. Greater changes were observed in the non-recovery group at all subsites (**Table 2**).

**Table 2:** Comparison of degeneration ratios between the groups.

	Recovery group	Nonrecovery group	P-value^a)^
Degeneration ratios (%) at the first ENoG			
Frontalis	30.9 ± 23.9	37.4 ± 26.6	0.444
Orbicularis oculi	31.8 ± 25.2	37.8 ± 34.5	0.589
Nasalis	31.6 ± 28.0	39.7 ± 30.3	0.410
Orbicularis oris	36.0 ± 35.4	55.5 ± 33.5	0.109
Degeneration ratios (%) at the second ENoG			
Frontalis	37.7 ± 23.2	63.0 ± 30.6	**0.006**
Orbicularis oculi	41.0 ± 26.6	66.8 ± 30.8	**0.010**
Nasalis	49.2 ± 23.6	71.2 ± 30.7	**0.016**
Orbicularis oris	48.0 ± 29.7	78.3 ± 30.2	**0.005**

BP, Bell’s palsy; RHS, Ramsay-Hunt syndrome; ENoG, Electroneuronography Frontalis; OC, Orbicularis oculi; N, Nasalis; OR, Orbicularis oris. ^a)^by Independent t-test

**Fig 3:**
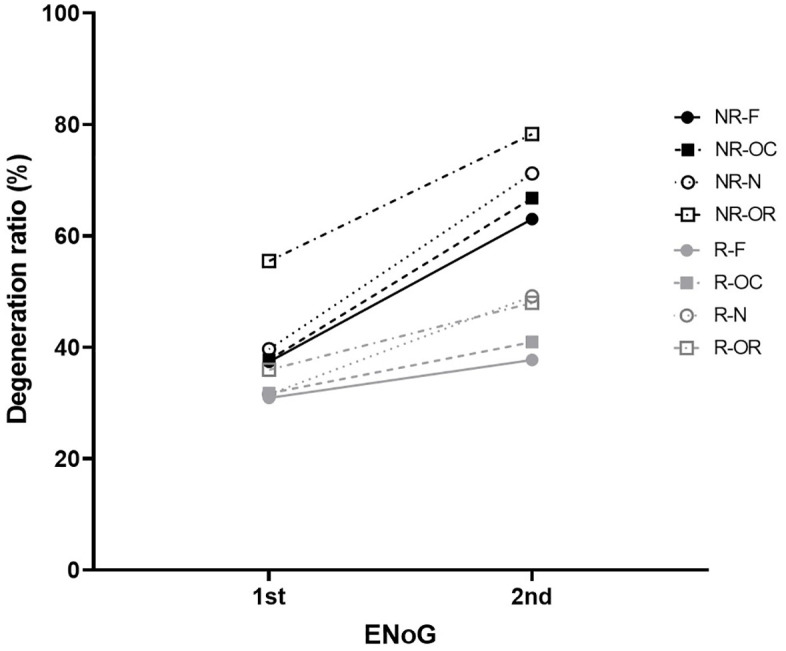
Degeneration ratios according to the testing time and subsites.

Degeneration ratios according to the subsite were compared. The values followed the descending order of the orbicularis oris, nasalis, orbicularis oculi, and frontalis muscles in all tests, except for the second test in the recovery group. This pattern corresponded to the anatomical sequence from the lower to upper regions of the face (**Fig 3**).

### Possible predictors of early recovery

A binary logistic regression analysis was performed to identify the most accurate predictor of early recovery from facial paralysis. Variables, including sex, age, diagnosis, and degeneration ratios of each site from the first and second ENoGs, were analyzed. Among the variables, the degeneration ratio of the orbicularis oris measured in the second ENoG was the only factor associated with early recovery from facial paralysis (odds ratio [OR], 0.961; p = 0.014). The Hosmer–Lemeshow test yielded a p-value of 0.114, indicating that the model was a good fit (**Table 3**).

**Table 3:** Binary logistic regression analysis to identify the predictors of early recovery from facial paralysis.

Variables	Odds ratio	P-value
Age		0.328
Sex		0.968
Diagnosis (BP or RHS)		0.355
**Degeneration ratios at the first ENoG**		
Frontalis		0.870
Orbicularis oculi		0.753
Nasalis		0.715
Orbicularis oris		0.723
**Degeneration ratios at the second ENoG**		
Frontalis		0.123
Orbicularis oculi		0.306
Nasalis		0.501
Orbicularis oris	**0.961**	**0.014**

BP, Bell’s palsy; RHS, Ramsay-Hunt syndrome; ENoG, Electroneuronography

**Fig 4:**
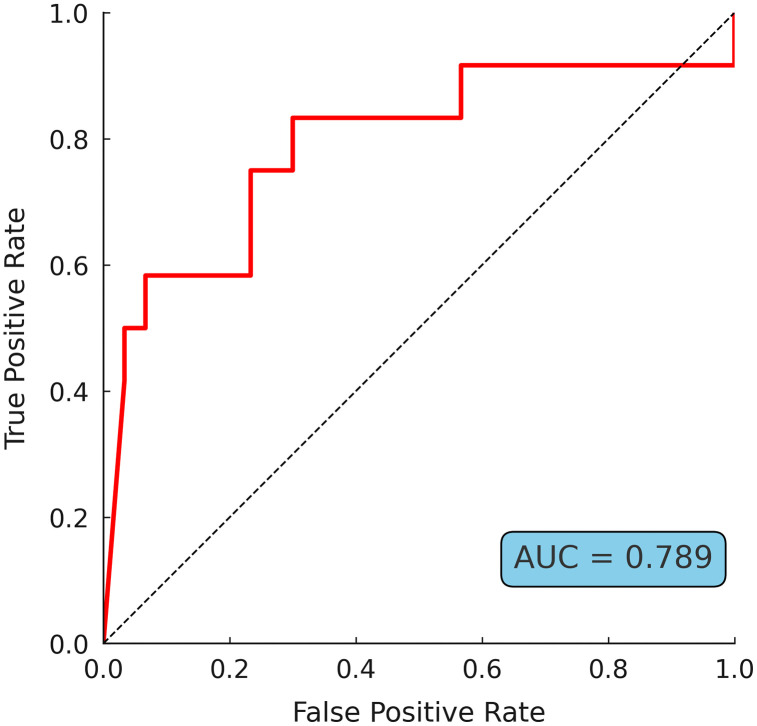
Receiver operating characteristic curve for predicting early recovery from the facial paralysis of orbicularis oris muscle at second electroneuronography measurement.

We conducted an ROC curve analysis to assess the degree to which ENoG measures could predict early recovery from facial paralysis. The degeneration ratios of the four subsites in the first and second ENoGs were analyzed. The AUC for all ENoG measurements from the first test was < 0.7, indicating that the measures were unsuitable for predicting early recovery. However, for the second test, the AUC > 0.7 at all subsites, with the highest value observed in the orbicularis oris (AUC = 0.789; **Table 4**; **Fig 4**). The optimal cutoff for the orbicularis oris was 67.9%, with a sensitivity of 83.3% and a specificity of 70.0%.

**Table 4:** Predictive ability of ENoG results using the receiver operating characteristic curve analysis.

Variables	AUC
**Degeneration ratios at the first ENoG**	
Frontalis	0.580
Orbicularis oculi	0.539
Nasalis	0.601
Orbicularis oris	0.661
**Degeneration ratios at the second ENoG**	
Frontalis	0.727
Orbicularis oculi	0.733
Nasalis	0.737
Orbicularis oris	0.789

AUC, area under curve; ENoG, Electroneuronography.

The degeneration ratio of the orbicularis oris, measured ~2 weeks post-onset, was the best predictor of early recovery from facial paralysis, and a degeneration ratio >67.9% was associated with a poor prognosis.

### Prediction of early recovery using the ENoG results

After identifying the best predictor, we further analyzed whether early recovery could be predicted using ENoG values measured at an early time point or those measured at other subsites. The analysis was conducted by dividing the entire patient population into two groups according to specific degeneration ratio cutoff. For instance, when the cutoff was set at 60%, patients were grouped as <60% or ≥60%, and the proportions of early recovery were compared between the two groups. A cutoff was considered meaningful if the difference in recovery rates between groups was statistically significant.

In the first ENoG, a significant difference in recovery was observed when the degeneration ratios of the frontalis were 80% and 90%, and when those of the orbicularis oculi were 60% and 70%. In the results of the second ENoG, a difference was observed in the proportion of recovery rates when the degeneration ratios of the frontalis were 50%, 70%, 80%, and 90%, those of the orbicularis oculi and nasalis were ≥60%, and that of the orbicularis oris was ≥ 50% degeneration ratio (**Table 5**).

**Table 5:** Comparisons of the proportions of recovery rate according to the degeneration ratios, ENoG measurement time, and ENoG measurement sites.

	10%	30%	50%	60%	70%	80%	90%
Degeneration ratios at 1st ENoG							
Frontalis	0.696	0.172	0.463	0.699	0.063	**0.019**	**0.019**
Orbicularis oculi	1.000	0.495	0.141	**0.049**	**0.046**	0.063	0.063
Nasalis	0.464	0.172	0.292	0.699	0.387	0.063	0.063
Orbicularis oris	0.277	0.139	0.499	0.174	0.117	0.406	0.406
Degeneration ratios at 2nd ENoG							
Frontalis	0.298	0.282	**0.008**	0.067	**0.001**	**<0.001**	**<0.001**
Orbicularis oculi	0.651	0.128	0.078	**0.032**	**0.020**	**0.014**	**0.014**
Nasalis	1.000	0.402	0.277	**0.011**	**0.001**	**0.004**	**0.004**
Orbicularis oris	0.655	0.128	**0.031**	**0.013**	**0.001**	**0.001**	**0.001**

ENoG, Electroneuronography.

Because it is more difficult to expect recovery at higher degeneration ratios, it was regarded as unreliable if significant differences were not found continuously according to the degeneration ratios. Ultimately, we concluded that early recovery cannot be achieved when the degeneration ratio of the frontalis is ≥ 80% in the initial ENoG. When the degeneration ratio of the frontalis was ≥ 70%, those of the orbicularis oculi and nasalis were ≥60%, and that of the orbicularis oris was ≥ 50% in the second ENoG, early recovery could not be expected.

## Discussion

The main findings of this study can be summarized as follows: first, ENoG conducted 2–3 weeks after the onset of paralysis is more useful for predicting early recovery from facial paralysis than ENoG conducted within the first week. Second, the degeneration ratios of the orbicularis oris measured 2–3 weeks post-onset provide the most accurate prediction of early recovery from facial paralysis. Finally, regardless of the subsite, when the degeneration ratios measured using ENoG 2–3 weeks post-onset exceed 60%, a lower recovery rate is expected.

When a peripheral nerve is damaged, Wallerian degeneration occurs 24–36 h after the injury [18], and regeneration starts approximately 3 weeks post-injury [19]. Owing to this mechanism, early ENoG may underestimate nerve damage due to its failure to reflect Wallerian degeneration, and late ENoG may also underestimate nerve damage as it reflects regeneration. Therefore, it is recommended to perform ENoG within 3–21 days of disease onset [9]. However, even within the recommended time period, ENoG results vary depending on the timing [20,21]. Bae et al. reported that ENoG conducted 5–8 days after onset was appropriate, attributing this to the time required for Wallerian degeneration to progress [22]. Similarly, Haginomori et al. suggested that ENoG conducted between 7 and 10 days post-onset was the most useful [21]. In this study, ENoG conducted 14.8 ± 3.0 days post-onset was more accurate in predicting early recovery than that conducted 4.8 ± 2.5 days post-onset. This finding underscores the importance of performing ENoG at an appropriate time, including during the Wallerian degeneration process; however, there are some differences in timing in the literature. Further research is needed to determine the optimal testing time.

Although the preferred testing time was identified, we also attempted to determine which ENoG site is the most helpful in predicting early recovery. This study revealed that the orbicularis oris demonstrated the highest sensitivity, which differs from the results of previous studies. Woo et al. reported that, in 37 patients with severe facial paralysis, the nasalis provided the most accurate prediction of prognosis 2 months post-onset, based on ENoG measurements conducted 2–4 weeks after onset [23]. Similarly, Kim et al. compared the prognosis at 6 months in 81 patients with facial paralysis using degeneration ratios of the orbicularis oculi and nasalis and concluded that the nasalis was a better predictor [24]. These studies explained the superiority of the nasalis in predicting the recovery of anatomical structures. According to most literature and anatomy textbooks, the frontalis muscle is innervated by the temporal branch, the orbicularis oculi muscle is double-innervated by the temporal and zygomatic branches, the nasalis muscle is innervated by the buccal branch, and the orbicularis oris muscle is double-innervated by the buccal and mandibular branches [25,26]. Among the facial nerve branches, the buccal branch has the most variable pathways and the greatest number of interconnections with other branches [27]. For this reason, the authors argued that the buccal branch most comprehensively represents facial nerve function; thus, the degeneration ratio measured in the nasalis muscle is the most valuable in predicting the prognosis of facial paralysis.

However, in this study, orbicularis oris was more sensitive to prognosis than the nasalis. Although both the muscles are innervated by the buccal branch, the observed differences can be attributed to several factors. The first factor is the difference in skin characteristics. Both the nasalis and orbicularis oris are superficial facial muscles [28], and skin thickness significantly affects the accuracy of electrophysiological tests. The conductivity of the skin is closely related to the thickness of the epidermis [29]; the epidermis of the orbicularis oris is thinner than that of the nasalis muscle [30]. In fact, one study reported that the orbicularis oris muscle shows greater amplitude and shorter latency than the nasalis muscle, indicating a relatively higher conductivity [31]. This could explain the higher conductivity and accuracy observed for orbicularis oris. Second, electrode placement must be considered. In standard ENoG, electrodes are typically placed on the nasolabial fold, not on the nasalis muscle, and the angle of the mouth. The muscles forming the nasolabial fold, such as the levator labii superioris, zygomatic major/minor, and zygomatic risorius, receive dual innervation from both the buccal and zygomatic branches. By contrast, the orbicularis oris is double-innervated by the buccal and mandibular branches. However, its lower portion is mainly supplied by the mandibular branch, whereas the lateral portion, particularly at the angle of the mouth, is predominantly supplied by the buccal branch. Therefore, under typical ENoG electrode placement, the orbicularis oris is expected to be more affected by the buccal branch than the nasalis muscle (nasolabial fold). Woo et al. also identified the orbicularis oris as the most reliable predictor in ROC curve analysis [23], supporting the findings of this study. Third, differences in equipment and participants may have contributed to the inconsistent results. The results are influenced by the device or size of the electrodes [32]. In addition, differences in factors such as ethnicity, severity of facial paralysis, and timing of the test may account for the discrepancies in the results.

In this study, we identified the preferred testing time and subsites for predicting early recovery from facial paralysis as well as attempted to determine whether early recovery could be predicted using ENoG values measured at an early time point or those measured at other subsites. This could be helpful in clinical practice when the testing time or subsites are limited.

According to the analysis, early recovery was difficult when the degeneration ratio of the frontalis muscle was ≥ 80% within 1 week of onset, and the degeneration ratios of all muscles were approximately ≥60% 2–3 weeks after onset. This finding is consistent with those reported in previous studies. Esslen reported that degeneration ratios below 50% were associated with a favorable prognosis [33], whereas Inamura stated that a degeneration ratio below 60% was necessary for recovery within 1 month [34]. Similarly, Haginomori et al. reported that patients with degeneration ratios below 40% fully recovered within 1 month, and those with rates between 40% and 80% recovered within 4 months [35]. However, long-term follow-up studies of 6 months to 3 years often used a 90% degeneration ratio as the recovery threshold [16,36,37]. Considering that nerve regeneration progresses at approximately 1 mm per day [38], it can be interpreted that, over time, patients with degeneration ratios between 60% and 90% show higher recovery rates. Nevertheless, predicting early recovery from facial paralysis is important, as prolonged facial paralysis can impair confidence and social interactions [39], making it essential for patients to reintegrate into social life as quickly as possible.

A notable observation in this study is that the degeneration ratios were higher in the lower facial muscles than in the upper facial muscles. This difference may be attributed to the physical characteristics of these nerves. Branches of the facial nerve are generally longer, thicker, and have fewer subdivisions in the zygomatic and buccal branches than in the temporal branch [27]. Idiopathic facial paralysis is primarily caused by compression injuries, and larger nerve diameters are more prone to compression-induced damage, according to Laplace’s law [40]. Similarly, optic nerves with larger diameters exhibit faster degeneration after injuries [41].

In this study, no significant difference was observed in the recovery rates between BP and RHS. However, the diagnosis is not a predictor of early recovery. It is generally known that RHS has a worse prognosis than BP [42]. We consider this discrepancy to be due to the small and biased number of patients, as there were only seven patients with RHS. A sufficiently large sample size may yield different results.

Another interesting finding is the predominance of right-sided facial paralysis. This could also be attributed to the small sample size, as a large-scale study involving 2570 patients found no significant difference in the incidence between the two sides [43]. However, a previous study reported that patients with right-sided facial paralysis had a significantly worse psychological status and social interactions [44]. It is possible that patients with right-sided facial paralysis are more likely to seek medical attention, resulting in more patients with right-sided facial paralysis in the present study.

This study has some limitations. First, it lacked long-term follow-up data. This study aimed to predict early recovery; therefore, the follow-up period was short. However, considering that the facial nerve may take 12–18 months to regenerate [45], the lack of a comparison of long-term outcomes is a limitation. Second, this study did not consider underlying diseases or lifestyle factors. Hypertension, diabetes, and dyslipidemia affect the severity and prognosis of facial paralysis [46]. Although the results are inconsistent, alcohol consumption and smoking have been suggested as risk factors for facial paralysis [47]. However, the present study lacked these data. Third, the study did not include a sufficient number of patients. The lack of differences in the recovery rates between patients with BP and RHS, as well as the higher incidence of right-sided paralysis, may have been influenced by the sample size.

Despite these limitations, this study highlights the potential of using ENoG as a simple, outpatient-based tool to predict early recovery from acute peripheral paralysis by providing preferred testing times and measurement sites. It also highlights the importance of the buccal branch in representing the facial nerve and provides clues for interpreting the results according to the location of the electrode. Given the individual and social impacts of facial paralysis, predicting early recovery is crucial for reassuring patients, providing better treatment, and encouraging their early reintegration into society. Furthermore, the findings of this study may assist clinicians in counseling patients regarding their expected recovery trajectory during the early stage of paralysis, potentially reducing anxiety and improving adherence to treatment. Future prospective multicenter studies with larger cohorts are warranted to validate these results and to establish standardized ENoG-based prognostic models. This study is clinically significant and expected to improve the diagnosis and management of this condition.

## Supporting information

S1 File De-identified raw data used for all analyses in the study. (XLSX)
